# Supplementary material for: Macrophages Upregulate Estrogen Receptor Expression in the Model of Obesity-Associated Breast Carcinoma
Source: Cells. 2022 Sep 12;11(18):2844. doi: 10.3390/cells11182844 (PMC9496942; doi:10.3390/cells11182844)
Supplement: Supplementary file 1 [file cells-11-02844-s001.zip › cells-1872749-supplementary Figure S3.pdf]

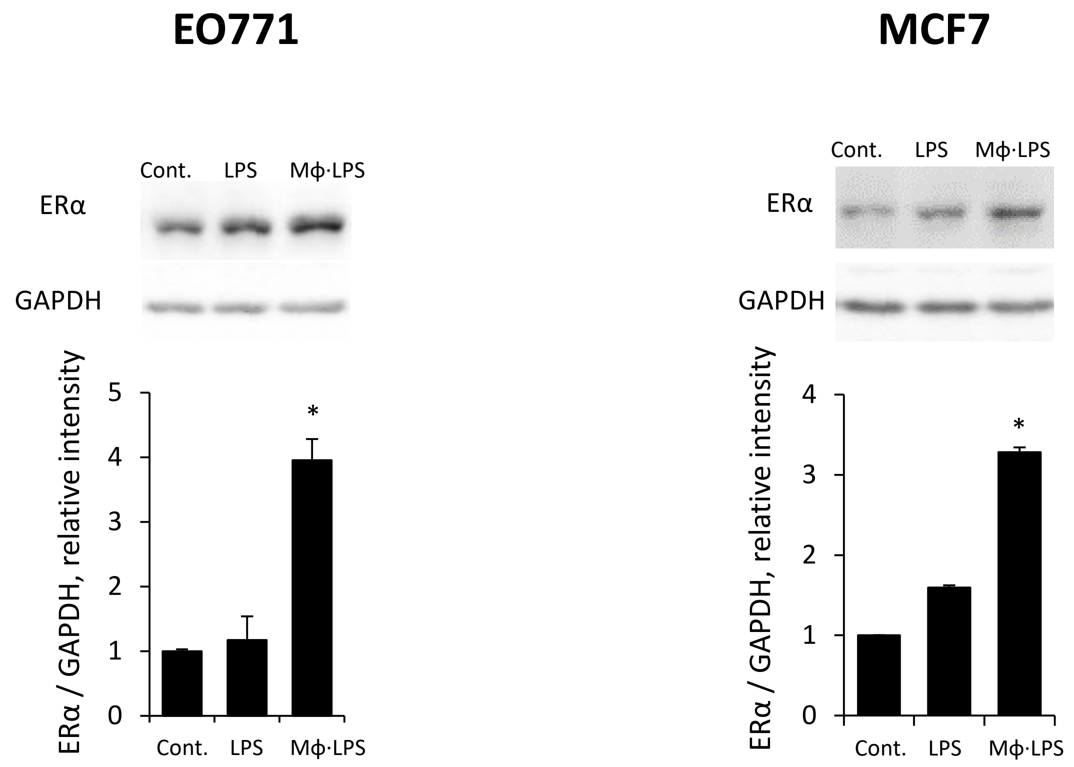

Supplementary Figure S3. E0771 (left) and MCF7 (right) cells were either remained untreated (Cont.) or incubated (24 h, 37°C) with LPS or medium conditioned by macrophages stimulated by LPS at concentration 0.1 ng/ml (Mϕ·LPS). ER*α* protein levels were determined by immunoblotting (top) and quantified using ImageJ software (bottom). Intensity ratio for ER*α* /GAPDH is shown. The data are representative of at least 3 independent experiments; error bars represent ±SD .*p<0.007.
