# Supplementary material for: Macrophages Upregulate Estrogen Receptor Expression in the Model of Obesity-Associated Breast Carcinoma
Source: Cells. 2022 Sep 12;11(18):2844. doi: 10.3390/cells11182844 (PMC9496942; doi:10.3390/cells11182844)
Supplement: Supplementary file 1 [file cells-11-02844-s001.zip › Supplementary fig S1.pptx]

## Slide 1
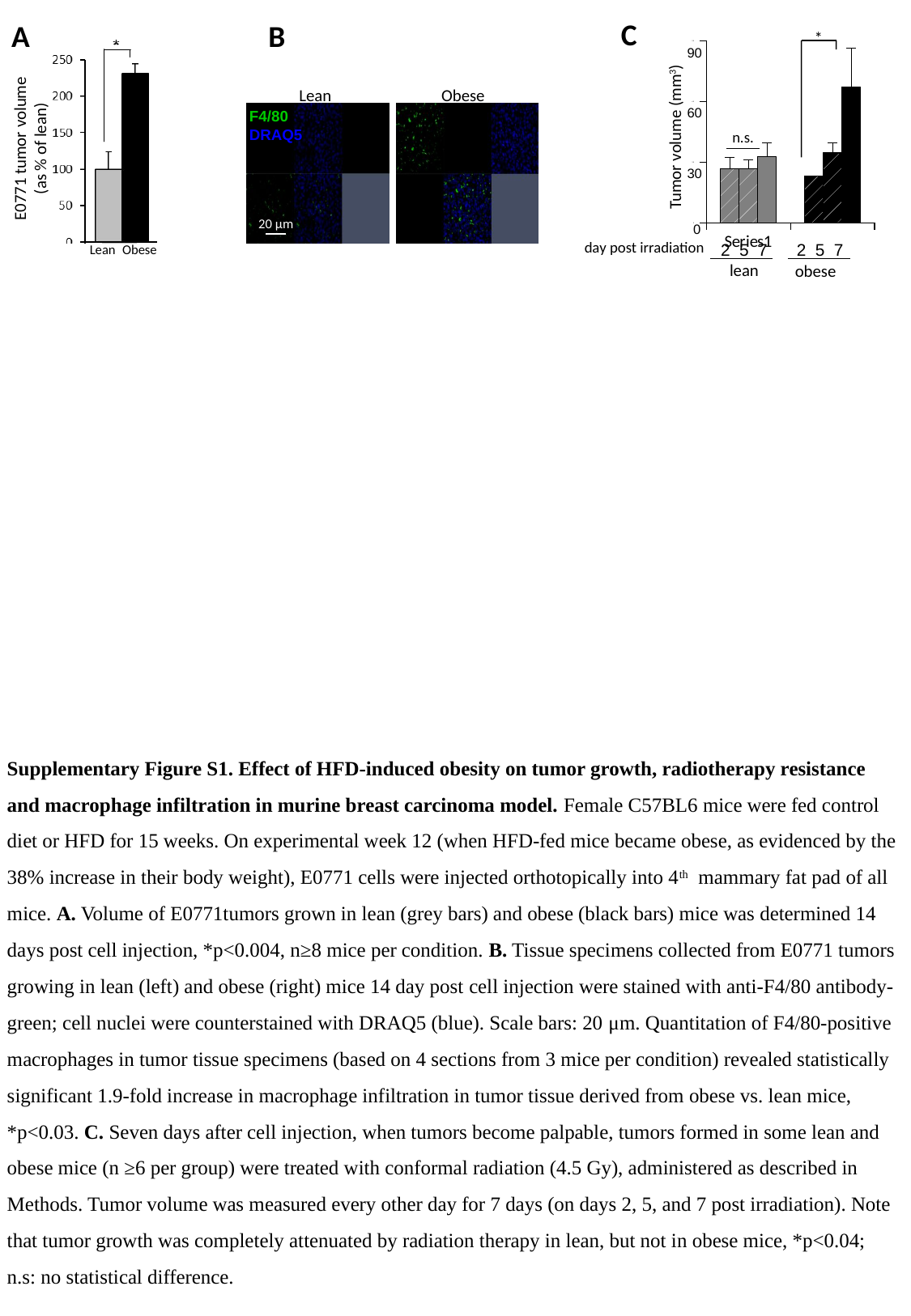

C
B
A
*
### Chart
| Category | | | |
|---|---|---|---|
| | 26.51388888888889 | 26.645833333333332 | 32.59027777777778 |
| | 22.9375 | 34.50625 | 67.1484375 |90
Lean
Obese
60
F4/80
DRAQ5
Tumor volume (mm3)
n.s.
E0771 tumor volume
(as % of lean)
30
20 µm
0
day post irradiation
2 5 7
2 5 7
Lean
Obese
lean
obese
Supplementary Figure S1. Effect of HFD-induced obesity on tumor growth, radiotherapy resistance and macrophage infiltration in murine breast carcinoma model. Female C57BL6 mice were fed control diet or HFD for 15 weeks. On experimental week 12 (when HFD-fed mice became obese, as evidenced by the 38% increase in their body weight), E0771 cells were injected orthotopically into 4th mammary fat pad of all mice. A. Volume of E0771tumors grown in lean (grey bars) and obese (black bars) mice was determined 14 days post cell injection, *p<0.004, n≥8 mice per condition. B. Tissue specimens collected from E0771 tumors growing in lean (left) and obese (right) mice 14 day post cell injection were stained with anti-F4/80 antibody- green; cell nuclei were counterstained with DRAQ5 (blue). Scale bars: 20 μm. Quantitation of F4/80-positive macrophages in tumor tissue specimens (based on 4 sections from 3 mice per condition) revealed statistically significant 1.9-fold increase in macrophage infiltration in tumor tissue derived from obese vs. lean mice, *p<0.03. C. Seven days after cell injection, when tumors become palpable, tumors formed in some lean and obese mice (n ≥6 per group) were treated with conformal radiation (4.5 Gy), administered as described in Methods. Tumor volume was measured every other day for 7 days (on days 2, 5, and 7 post irradiation). Note that tumor growth was completely attenuated by radiation therapy in lean, but not in obese mice, *p<0.04; n.s: no statistical difference.
