# Supplementary material for: Macrophages Upregulate Estrogen Receptor Expression in the Model of Obesity-Associated Breast Carcinoma
Source: Cells. 2022 Sep 12;11(18):2844. doi: 10.3390/cells11182844 (PMC9496942; doi:10.3390/cells11182844)
Supplement: Supplementary file 1 [file cells-11-02844-s001.zip › Supplementary fig S2.pptx]

## Slide 1
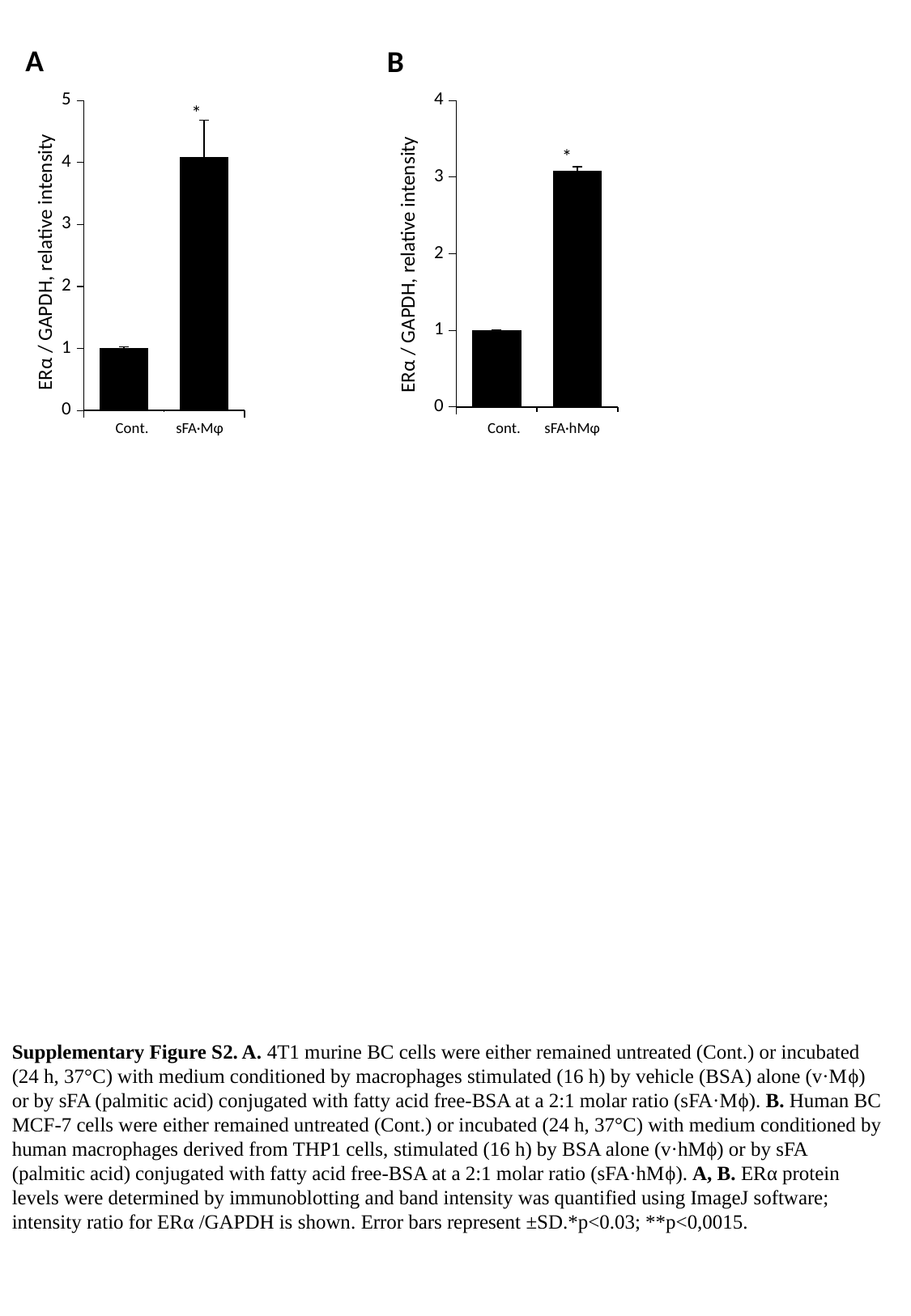

A
B
### Chart
| Category | |
|---|---|
| Cont | 1.0 |
| Mφ C16 | 4.0788069201025206 |
### Chart
| Category | 1 |
|---|---|
| Untreated | 1.0 |
| sFA | 3.0758067443113064 |*
*
ERα / GAPDH, relative intensity
ERα / GAPDH, relative intensity
Cont. sFA·Mφ
Cont. sFA·hMφ
Supplementary Figure S2. A. 4T1 murine BC cells were either remained untreated (Cont.) or incubated (24 h, 37°C) with medium conditioned by macrophages stimulated (16 h) by vehicle (BSA) alone (v·Mϕ) or by sFA (palmitic acid) conjugated with fatty acid free-BSA at a 2:1 molar ratio (sFA·Mϕ). B. Human BC MCF-7 cells were either remained untreated (Cont.) or incubated (24 h, 37°C) with medium conditioned by human macrophages derived from THP1 cells, stimulated (16 h) by BSA alone (v·hMϕ) or by sFA (palmitic acid) conjugated with fatty acid free-BSA at a 2:1 molar ratio (sFA·hMϕ). A, B. ERα protein levels were determined by immunoblotting and band intensity was quantified using ImageJ software; intensity ratio for ERα /GAPDH is shown. Error bars represent ±SD.*p<0.03; **p<0,0015.
