# Supplementary material for: Macrophages Upregulate Estrogen Receptor Expression in the Model of Obesity-Associated Breast Carcinoma
Source: Cells. 2022 Sep 12;11(18):2844. doi: 10.3390/cells11182844 (PMC9496942; doi:10.3390/cells11182844)
Supplement: Supplementary file 1 [file cells-11-02844-s001.zip › Supplementary Figure S4.pdf]

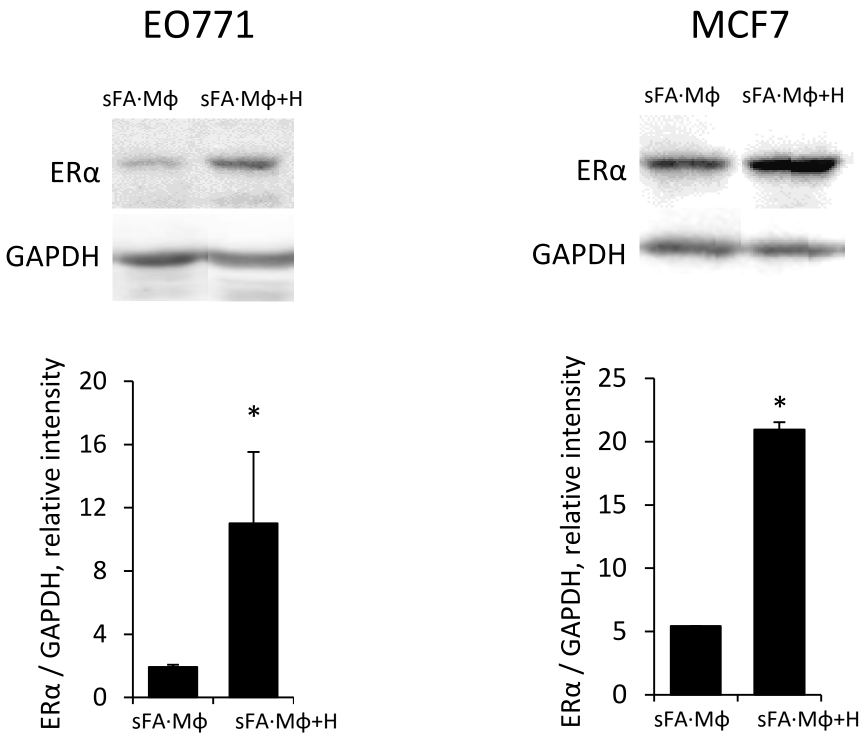

Supplementary Figure S4. Heparanase augments ability of sFA-stimulated macrophages to upregulate ERα expression in BC cells. E0771 (left) and MCF7 (right) BC cells were incubated for 16 h with medium conditioned (24 h, 37°C) by primary *wt* macrophages, stimulated by sFA in the absence (sFA·Mϕ), or presence of active recombinant heparanase (sFA·Mϕ+H). **Top panels:** lysates of E0771 (**A**) and MCF7 (**B**) cells were immunoblotted using antibody specific for ERα and GAPDH. **Bottom panels:** The band intensity was quantified using ImageJ software, error bars represent ±SD .*$p<0.02$.
